# Flexible carbon nanotube/mono-crystalline Si thin-film solar cells

**DOI:** 10.1186/1556-276X-9-514

**Published:** 2014-09-20

**Authors:** Huanhuan Sun, Jinquan Wei, Yi Jia, Xian Cui, Kunlin Wang, Dehai Wu

**Affiliations:** 1 Key Laboratory for Advanced Materials Processing Technology of Education Ministry; State Key Laboratory of New Ceramic and Fine Processing, School of Materials Science and Engineering, Tsinghua University, Beijing 100084, China; 2 College of Materials Science and Engineering, Beijing University of Chemical Technology, Beijing 100029, China; 3 Department of Mechanical Engineering, Tsinghua University, Beijing 100084, China

**Keywords:** Carbon nanotubes, Si thin film, Solar cell, Heterojunction

## Abstract

Flexible heterojunction solar cells were fabricated from carbon nanotubes (CNTs) and mono-crystalline Si thin films at room temperature. The Si thin films with thickness less than 50 μm are prepared by chemically etching Si wafer in a KOH solution. The initial efficiency of the thin-film solar cell varies from approximately 3% to 5%. After doping with a few drops of 1 M HNO_3_, the efficiency increases to 6% with a short-circuit current density of 16.8 mA/cm^2^ and a fill factor of 71.5%. The performance of the solar cells depends on the surface state and thickness of Si thin films, as well as the interface of CNT/Si. The flexible CNT/Si thin-film solar cells exhibit good stability in bending-recovery cycles.

## Background

Low-cost, high-efficiency, and flexible solar cells have attracted great attention due to the increasing energy demands [1-4]. Currently, crystalline Si-based solar cells dominate the photovoltaic market because of their relatively high module efficiency, high stability, and well-established process technology. Mono-crystalline Si thin films with a thickness less than 50 μm are thus expected for making a high-efficiency solar cell, so as to reduce the materials cost. However, it is difficult to manufacture crystalline Si thin films in large quantity by using traditional wire cutting process due to their brittleness. Recently, mono-crystalline Si ribbons and thin films with 2 ~ 50 μm in thickness were fabricated through a wet chemical etching process from bulk Si wafers [5-8]. Flexible mono-crystalline Si solar cells were thus fabricated by making p-n junction through high-temperature doping. Up to now, the efficiency of these crystalline Si-based flexible solar cells is approximately 6% to 13.7% [5-8], which is higher than that of conjugated polymer cells and amorphous Si thin-film tandem solar cells [3,4,9] but still lower than that of commercial bulk Si solar cell modules. Furthermore, these Si thin-film solar cells are still required to make p-n junction through doping at high temperature, which is a costly process and also increases the risk of damage to the Si thin films.

Recently, we developed a heterojunction solar cell by combining carbon nanotubes (CNTs) and bulk Si wafer [10]. The CNT/Si heterojunction solar cell has aroused great interests because of its simple process and relatively high efficiency [11-17]. We have successfully improved the efficiency of the solar cells from an initial value of approximately 6% to 13.8% by HNO_3_ doping and further to 15% by coating with a thin layer of nano-TiO_2_ particles [11-13]. The CNT/Si solar cells with high efficiency above 10% were also fabricated in other groups, showing the promising photovoltaic devices with a simple structure [14-18]. Most of these researches focused on improving the efficiency by chemical doping, improving the alignment of CNTs, or using p-type semiconducting tubes. Amorphous Si thin films were also used to construct CNT/Si cells in order to cut down the materials cost [19]. Due to the high recombination rate of the electron-hole pairs within the amorphous Si thin film, the CNT/Si cells made from amorphous Si have very poor efficiency. In order to reduce the electron transport distance and recombination rate of electron-hole pairs within Si side, cut down the materials cost, as well as meet the future demands of flexible energy devices, we fabricate flexible CNT/Si solar cells with moderate efficiency from ultrathin mono-crystalline Si films.

## Methods

**Figure 1:**
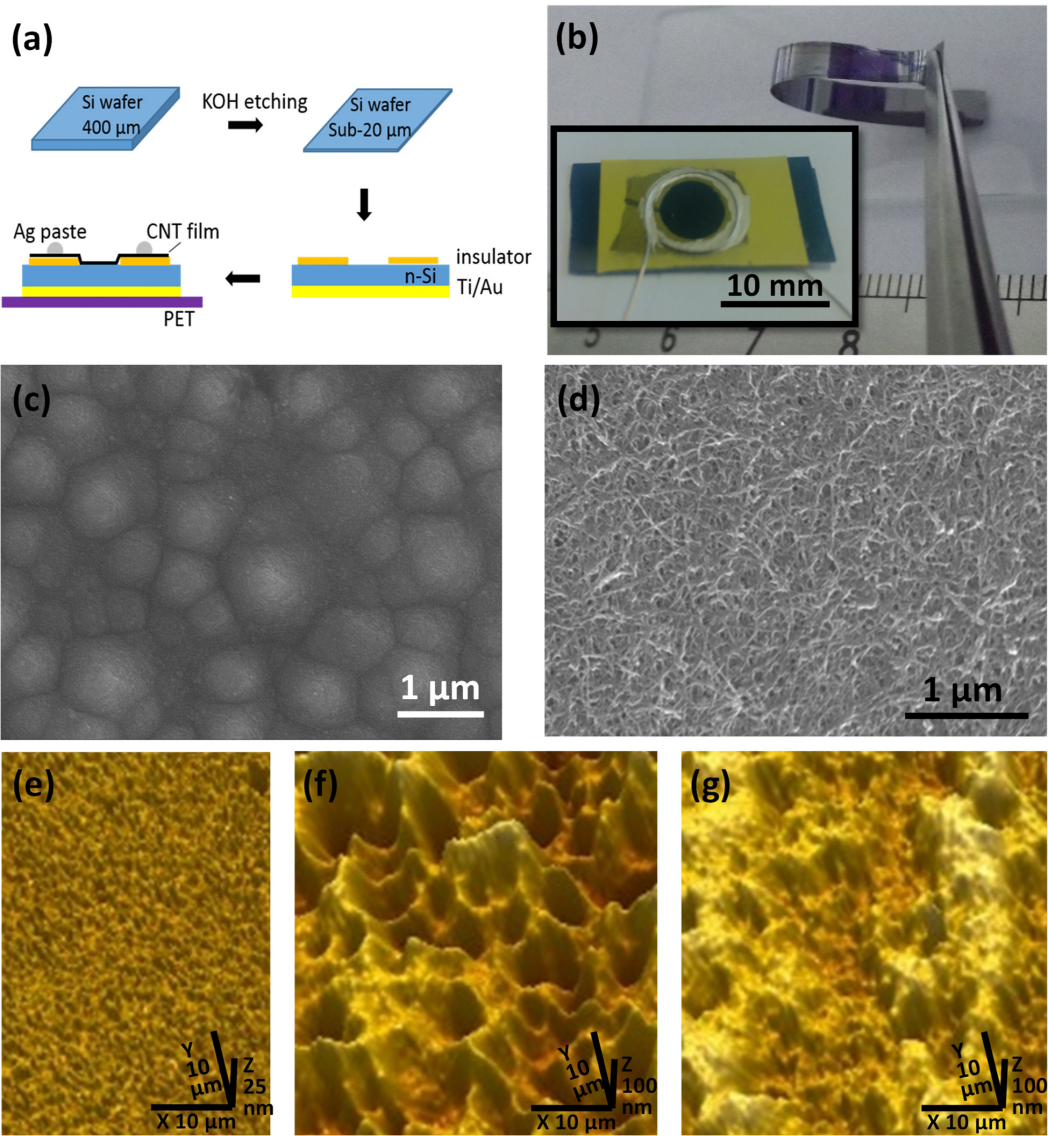
**Schematic illustration of the CNT/Si thin-film solar cell fabrication and sample images of Si films. (a)** Schematic illustration of the fabrication process of the CNT/Si thin-film solar cells. **(b)** Optical image of a large area of Si film with a thickness of 20 μm. Inset is a solar cell made from Si thin film. **(c)** SEM image of a Si thin film showing a textured surface. **(d)** SEM image of a CNT/Si thin-film solar cell. White light interferometry surface topography image of a starting Si wafer **(e)**, an etched Si thin film **(f)**, and a CNT/Si thin-film solar cell **(g)**.

CNT films (mixture of single-walled and double-walled tubes) were prepared by an improved chemical vapor deposition method [20]. The as-grown CNT films were purified by immersing in concentrated H_2_O_2_ (30 wt.%) and then in HCl (37 wt.%) solution for more than 48 h to remove amorphous carbon and catalyst particles. After rinsing by deionized water several times, the purified CNT films were expanded on water surface to form ultrathin films with a thickness of about 50 to 200 nm by adding a few drops of ethanol [21]. Figure 1a illustrates the fabrication process of CNT/Si thin-film solar cells. First, mono-crystalline Si thin films were prepared by chemical etching of n-type (100) Si wafers (4 in., 2 to 4 Ω cm, 400 ± 5 μm in thickness) in a 50 wt.% KOH solution at 80°C to 95°C according to a recent report [7]. The etched Si thin films were rinsed in deionized water several times to remove the residual KOH. Then, an insulating tape (approximately 150 μm in thickness) with a circular window of 6 mm in diameter is stuck on one side of the Si thin film. The tape can separate the front electrode from the Si thin film, protect the Si thin film from breaking, and ensure that light illustrates only on the CNT/Si heterojunction. A layer of Ti/Au thin film was then deposited on the other side of the Si thin film. A piece of purified CNT film was then transferred to the Si thin film on the side with tape. Finally, two silver wires were connected to the CNT film and Ti/Au film through silver paint, acting as front and back electrodes, respectively. To improve the efficiency of the solar cells, a few drops of 1 M HNO_3_ solution was added on the interface between CNTs and Si. After acid doping for several minutes, the residual acid was removed from the interface. The Si thin films were characterized and evaluated by scanning electron microscopy (SEM, Leo 1530, Carl Zeiss, Oberkochen, Germany) and white light interferometry (WLI, Rtec Optical Profilometer, Rtec Instruments, San Jose, CA, USA), respectively. The photovoltaic characteristics of the CNT/Si thin-film solar cells were tested under a solar simulator (Newport 91192-1000, Newport Corporation, Irvine, CA, USA) operating at AM 1.5.

## Results and discussion

The Si thin films were fabricated by wet chemical etching in a KOH solution from bulk Si wafers using the similar method reported in a recent paper [7]. During the etching, a large amount of gas was released from the Si surface. The etching rate reaches to approximately 180 μm/h when the temperature is kept at 90°C, which is faster than that of the recent report [7]. The thickness of the Si thin films is well controlled by the etching parameters, such as concentration of KOH, temperature, and etching time. Figure 1b shows an optical image of a Si thin film with a thickness of approximately 20 μm. The Si thin film can be bent to almost 180° by tweezers, showing a good flexible characteristic. Actually, the Si films are flexible and bendable when the thickness is less than approximately 50 μm. The inset of Figure 1b shows an optical image of a CNT/Si thin-film solar cell. Because of anisotropic etching, the Si thin films have pyramid-like texture on their surface (Figure 1c), which increases the roughness of the Si surface significantly. However, it is hard to observe such texture under SEM when the Si thin films are covered by CNTs (Figure 1d). It indicates that not all of the CNTs contact with the Si surface and some CNT bundles might bridge over of the pyramid-like peaks of the Si thin films.To further evaluate the surface roughness of the Si thin film, we performed WLI measurement to the different samples. Figure 1e is a WLI profile of the starting Si wafer, showing a roughness of 5 to 8 nm. For a Si thin film with a thickness of 20 μm, the roughness increases evidently to approximately 80 nm, which is more than ten times larger than that of the starting Si wafer (see Figure 1f). Thus, there are large amounts of dangling bonds on the rough Si surface. Although some wrinkles of the CNT film with a height of approximately 250 nm are also detected in the WLI profile, the roughness decreases evidently when the Si thin film is covered by a CNT film (see Figure 1g).

**Figure 2:**
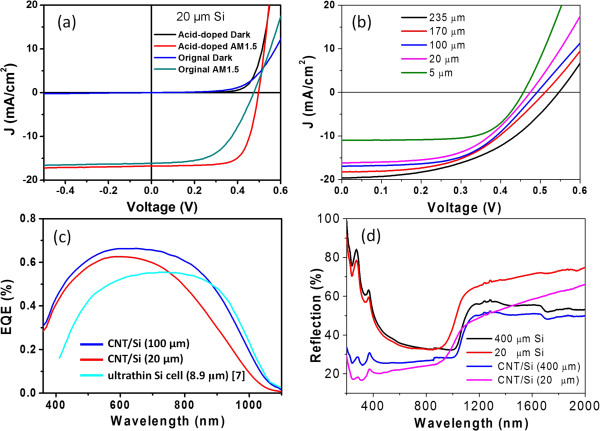
***J*-*V *curves, external quantum efficiency (EQE), and reflection spectra of a CNT/Si thin film. (a)** Dark and light *J*-*V* curves of a CNT/Si (20 μm) thin-film solar cell with and without HNO_3_ doping. **(b)** Light *J*-*V* curves of CNT/Si thin-film cells made from Si thin film with various thicknesses. **(c)** EQE of CNT/Si thin-film solar cells made from 100- and 20-μm-thick Si films. **(d)** Reflection spectra of Si thin film and corresponding CNT/Si solar cells.

Figure 2a shows dark and light *J*-*V* curves of a CNT/Si (20 μm) thin-film solar cell before and after 1 M HNO_3_ doping. The dark *J*-*V* curves show ultralow leakage current at reverse bias voltage. The ideality factor (*n*) calculated from dark *J*-*V* curves drops from 2.0 for original cell to 1.6 by HNO_3_ doping. The efficiency of the initial CNT/Si (20 μm) thin-film solar cell is only *η* = approximately 4.2%, which is lower than that we made from Si bulk wafer (approximately 7%) [12], but higher than that when an insulating tape is used to separate CNTs and Si [10]. The decrease of efficiency is derived mainly from the decline of short current density (*J*_sc_, 16.2 mA/cm^2^ vs approximately 26 mA/cm^2^) [12]. Due to the rough surface of the Si thin film and thick insulating tape, some CNT bundles might not contact with Si, leading to high surface recombination rate, high series resistance, and *J*_sc_ reduction. The interface between CNTs and Si thin film can be improved by HNO_3_ doping. After acid doping, the cell shows a moderate power conversion efficiency (*η*) of 6%, with an open-circuit voltage (*V*_oc_) of 495 mV, a short-circuit current density of 16.8 mA/cm^2^, and a fill factor (FF) of 71.5%, showing a significant effect of the heterojunction interface on the performance. The acid doping can reduce the series resistance and improve the interface between Si and CNTs by providing more charge transport path, resulting in a significantly improved FF and efficiency (see Table 1). The efficiency is almost as high as that of the mono-crystalline Si thin-film (8.9 μm) p-n junction solar cell made by high-temperature doping (6.2%) [7].

**Table 1:** Photovoltaic parameters of CNT/Si solar cells made from Si thin film with different thicknesses

**Si thickness (μm)**	***V***_**oc **_**(mV)**	***J***_**sc **_**(mA/cm**^**2**^**)**	**FF (%)**	***η *** **(%)**	***n***
235	540	19.7	47.9	5.1	1.9
170	510	18.2	49.7	4.6	2.3
100	506	17.0	52.5	4.5	2.2
20	472	16.2	56.5	4.2	1.9
5	456	11.0	68.0	3.4	1.8
20 (acid doping)	495	16.8	71.5	6.0	1.6

The photovoltaic performance of the CNT/Si thin-film solar cells depends not only on the interface of heterojunction but also on the thickness of the Si thin film. Figure 2b shows the light *J*-*V* curves of the CNT/Si solar cells made from Si thin film with various thicknesses. The photovoltaic parameters of the CNT/Si thin-film solar cells obtained from light *J*-*V* curves are given in Table 1. It is clear that both *V*_oc_ and *J*_sc_ decrease when the thickness of Si thin film reduces. When the thickness of Si films reduces from 235 to 20 μm, the *V*_oc_ decreases from 540 to 472 mV, while the *J*_sc_ decreases slowly from 19 to 16.6 mA/cm^2^. It is well known that if the surface passivation is not good enough, *V*_oc_ decreases with decreasing the wafer roughness because of surface recombination. The roughness increases as the etching time extends. So, it needs to reduce surface recombination rate by polishing Si surface so as to improve the efficiency.

Figure 2c shows external quantum efficiency (EQE) profiles of two CNT/Si thin-film solar cells with Si thicknesses of 20 and 100 μm, respectively. The EQE spectrum of the 20-μm-thick Si film cell is lower than that of the 100-μm-thick Si film, especially in the long-wavelength region (>700 nm), which is in good agreement with the light *J*-*V* curves. The reduction of EQE derives from increasing of light transmission in ultrathin Si film. It needs to be noted that the EQE of the CNT/Si solar cell 20-μm-thick Si films is higher than that of ultrathin Si cell with a thickness of 8.9 μm [7]. As an indirect bandgap semiconductor, it needs large thickness for crystalline Si film to completely absorb the light. It indicates that the *J*_sc_ of CNT/Si solar cells is also affected by optical absorption in crystalline Si thin film. It might be the main reason for the significant drop of *J*_sc_ from 16.2 to 11 mA/cm^2^ when the thickness of Si thin film reduces from 20 to 5 μm.Figure 2d shows the reflection spectra of the Si thin film and CNT/Si solar cells, respectively. The reflectance of the Si thin film is almost the same as that of the starting Si wafer at visible region but higher than that at infrared region (>1,100 nm). Figure 2d also shows a sharp increase of the reflectance at the wavelength above approximately 1,100 nm, corresponding to the bandgap of Si and CNT/Si. The bandgap of Si thin film shows a blueshift slightly to the original Si wafer according to the reflection spectra. When the Si thin film is covered by the CNT film, the reflectance declined evidently, which indicates that CNTs can enhance the light absorption at visible region.

**Figure 3:**
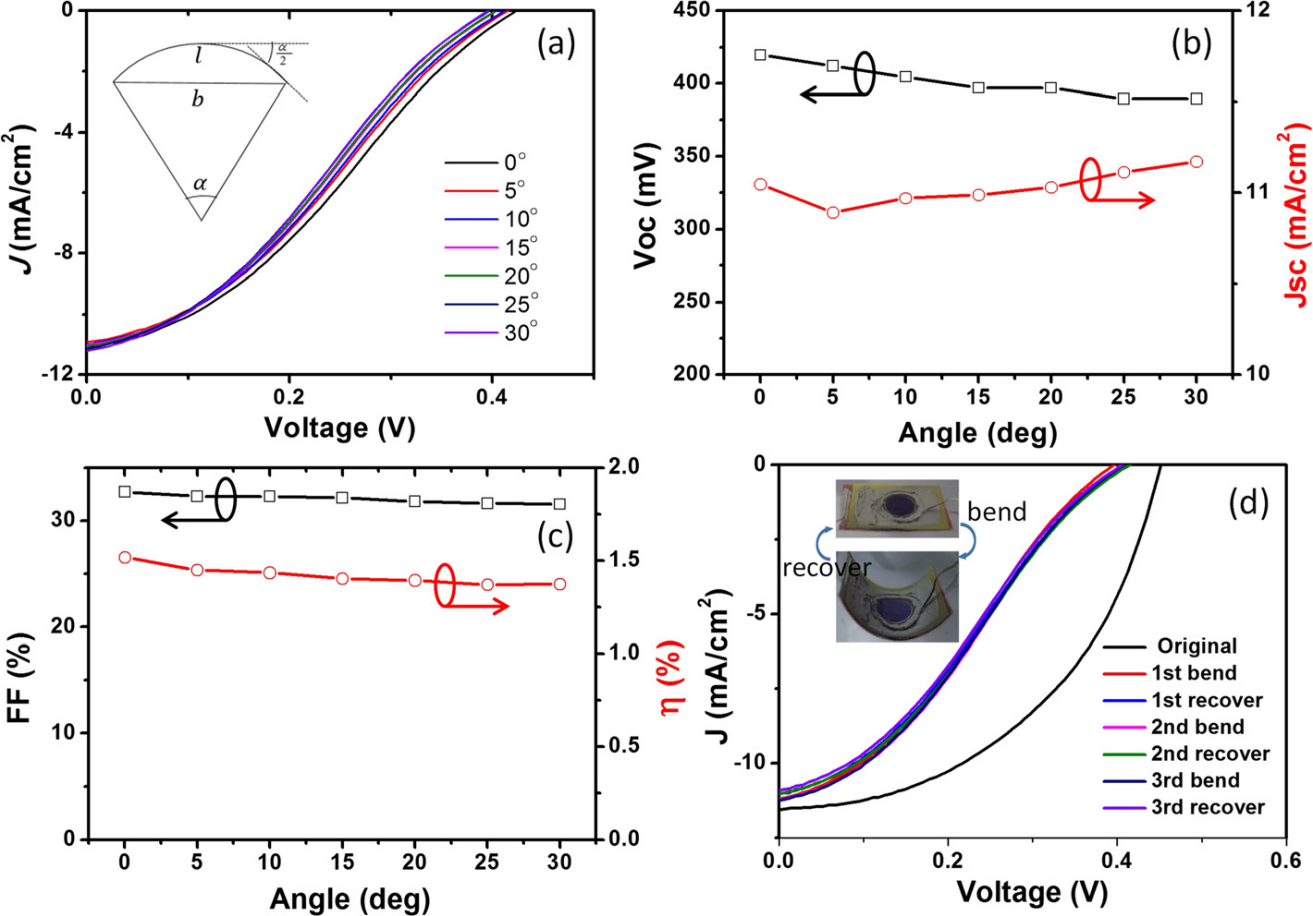
**Photovoltaic properties of a CNT/Si thin-film solar cell under bending. (a)***J-V* characteristics of a solar cell under bending. **(b)** Dependence of *V*_oc_ and *J*_sc_ on the bending angle. **(c)** Dependence of FF and *η* on the bending angle. **(d)** Light *J*-*V* curves of a CNT/Si thin-film solar cell with various bending-recovery cycles. Inset is the optical images of the cell under bending and recovery.

The CNT/Si thin-film solar cells are flexible and bendable. Figure 3a shows the light *J*-*V* curves of a CNT/Si (20 μm) thin-film solar cell at various bending angles (*α*). Some solar cells might break down when the bending angle exceeds a certain angle. We found that the maximum bending angles of the solar cells depend on the thickness and also on the connection between solar cells and the underneath substrate. For the ultrathin Si films with a thickness less than 20 μm, the maximum bending angle can exceed 50°. Figure 3b,c depicts that the photovoltaic parameters (*V*_oc_, *J*_sc_, FF, and *η*) depend on the bending angles. The *V*_oc_ of the CNT/Si solar cell decreases gently from 420 to 390 mV when the angle increases from 0° to 30°, while the *J*_sc_ increases slightly. The change of power conversion efficiency with the bending angle is similar to that of the FF. The *η* decreases slightly from 1.5% to 1.4% when the bending angle increases from 0° to 30°, showing a good stability at bending.

We also tested *J*-*V* curves of a CNT/Si thin-film solar cell under several bending and recovery cycles. An original CNT/Si solar cell (with an initial efficiency of *η* = 1.52%) is first bended to 30° and then recovered to original state. Figure 3d shows the light *J*-*V* curves under three bending-recovery cycles. For the first bending, the efficiency of the cell decreases from 2.5% to 1.4%. After the first bending, the light *J*-*V* curves almost overlap with each other in the three bending-recovery cycles, showing a good reliability of the flexible CNT/Si solar cells in bending and recovery cycles.

## Conclusions

In summary, flexible mono-crystalline Si thin films with a thickness varying from 5 to 50 μm were fabricated by wet chemical etching of bulk wafers in a KOH solution. The flexible CNT/Si heterojunction solar cells are made at room temperature from the CNT films and mono-crystalline Si thin film. The efficiency of the CNT/Si thin-film solar cells depends on the surface and thickness of Si thin films, as well as the interface of heterojunction. The CNT/Si thin-film solar cells show an initial efficiency of approximately 3% to 5% and reaches to 6% by HNO_3_ doping. The CNT/Si thin-film solar cells show a good stability in bending-recovery cycles.

## Competing interests

The authors declare that they have no competing interests.

## Authors’ contributions

HS, YJ, and JW carried out most of the experiments; XC prepared the CNT samples; HS and JW prepared and revised the manuscript. All of the authors discussed the results and commented on and approved the paper.

## Authors’ information

HS received his BS degree in Mechanical Engineering from Tsinghua University, China, in 2012. At the moment, he is pursuing his Master's Degree in Materials Science and Engineering at Tsinghua University. His research interests are in solar cells and nanomaterials.

JW received his BS and doctoral degree in Mechanical Engineering from Tsinghua University, China, in 1999 and 2004, respectively. He became a faculty member at the Department of Mechanical Engineering at Tsinghua University since 2006. He transferred to the School of Materials Science and Engineering at Tsinghua University in 2013. He has been working in the field of nanomaterials since 1998 and in the field of solar energy since 2006.

YJ received his BS and doctoral degree in Mechanical Engineering from Tsinghua University, China, in 2005 and 2011, respectively. Currently, he is a faculty member at the College of Materials Science and Engineering, Beijing University of Chemical Technology, China. His research interests are nanomaterials and solar energy.

XC received his BS degree in Mechanical Engineering from Tsinghua University, China, in 1999. At the moment, he is a technician working at the School of Materials Science and Engineering at Tsinghua University, China.

KW transferred from the Department of Mechanical Engineering to the School of Materials Science and Engineering at Tsinghua University in 2013. He is a professor and group leader of the Laboratory of Carbon Nanomaterials.

DW is a professor in the Department of Mechanical Engineering at Tsinghua University, China. He has been working in the field of carbon nanomaterials since 1992 and retired in 1999.
